# Glucose Intake Alters Expression of Neuropeptides Derived from Proopiomelanocortin in the Lateral Hypothalamus and the Nucleus Accumbens in Fructose Preference Rats

**DOI:** 10.1155/2017/6589424

**Published:** 2017-11-08

**Authors:** Guangfa Jiao, Guozhong Zhang, Haiying Wang, Weilin Zhao, Yanwei Cui, Yongjing Liu, Feng Gao, Fang Yuan, Yi Zhang

**Affiliations:** ^1^Department of Physiology, Hebei Medical University, Shijiazhuang 050017, China; ^2^Department of Human Sport Science, Hebei Institute of Physical Education, Shijiazhuang 050000, China; ^3^Hebei Key Laboratory of Forensic Medicine, Institute of Forensic Medicine of Hebei Medical University, Shijiazhuang 050017, China; ^4^Sports Institute, Hebei Normal University, Shijiazhuang 050040, China; ^5^Hebei Collaborative Innovation Center for Cardio-cerebrovascular Disease, Shijiazhuang 050000, China

## Abstract

To study the neuroendocrine mechanism of sugar preference, we investigated the role of glucose feeding in the regulation of expression levels of neuropeptides derived from proopiomelanocortin (POMC) in the lateral hypothalamus (LH) and nucleus accumbens (NAc) in fructose preference rats. Fructose preference rats were induced by using the lithium chloride backward conditioning procedure. The fructose preference was confirmed by the two-bottle test. The drinking behavior of rats was assessed by the fructose concentration gradient test. The preference of 10% glucose or 0.1% saccharine was assessed, and the expression levels of neuropeptides derived from POMC in the LH and the NAc in fructose preference rats were measured by Western blot analysis. Fructose preference rats displayed a greater fructose preference than control rats. Furthermore, fructose preference rats preferred glucose solution rather than saccharine solution, while control rats preferred saccharine solution rather than glucose solution. The expression levels of neuropeptides derived from POMC in the LH and the NAc were changed by glucose but not saccharine intake. In summary, the data suggests that glucose intake increases the expression of neuropeptides derived from POMC in the LH and the NAc in fructose preference rats.

## 1. Introduction

Obesity is prevalent in a large portion of the world's population, as a result of the abundance of palatable hypercaloric foods [1]. Food addiction, manifested by food preference and binge eating disorder (BED), is one of the causes of this global health problem [2]. In modern society, individuals with obesity are more likely to be addicted to hypercaloric food containing carbohydrates. High-calorie sugar and noncalorie artificial sweeteners are different addictive substances [3]. It remains unclear if the sweet taste or the calories in sugar induces the hedonic overeating that produces a reward in sugar preference.

A previous study has shown that reward and motivation of feeding is controlled by neural circuits and neuroendocrine signals [4]. The hypothalamus is a brain region that controls satiation and starvation [5] and maintains energy homeostasis. The regulatory pathway from the arcuate nucleus (ARC) to the lateral hypothalamus (LH) may be involved in severe hyperphagia and short-term control of feeding behavior [6]. The nucleus of the solitary tract (NTS), nucleus accumbens (NAc), and ventral tegmental area (VTA) in the nonhypothalamic system also play critical roles in the regulation of food intake and reward-related eating [7].

Proopiomelanocortin (POMC) is mainly expressed in the ARC and the NTS of the brainstem [8] and can be cleaved into multiple neuropeptides, such as *α*-melanocyte stimulating hormone (*α*-MSH), *β*-MSH, adrenocorticotropin (ACTH), *β*-endorphin (*β*-END), and *β*-lipotropin (*β*-LPH) [9]. POMC neurons in the ARC project and release *α*-MSH into many hypothalamic nuclei including LH [10]. Also, the POMC gene is expressed in the NAc reward system [11]. However, the function of POMC and the neuropeptides derived from POMC in the NAc, as well as the LH, is not well-understood in food addiction.

Melanocortin derived from POMC neurons is a well-characterized neuronal signal involved in the regulation of energy homeostasis. POMC neurons play an important role in the regulation of food intake [12] and are involved in cannabinoid-induced promotion of feeding [13]. POMC neurons are a key driver of ignition or cessation of feeding behavior. The dysregulation of the POMC system including POMC neurons and POMC-related neuropeptides plays a pivotal role in food addiction. We hypothesize that the POMC system in the NAc and LH is important in regulating sugar preference.

We used a rat model of fructose preference (conditioned stimulus) based on the theory of the backward conditioning procedure using lithium chloride (unconditioned stimulus) [14]. Fructose is the sweetest sugar among all naturally produced carbohydrates [15]. Preference for food or sugar solutions is due to the sweet flavor taste and postoral effect of sugar that may lead to food addiction [16]. In this study, we assessed the responses of fructose preference rats to different concentrations of fructose solution and to calorie (glucose) and noncalorie (saccharine) sweet solutions. We also determined the expression of neuropeptides derived from POMC in the LH and NAc in fructose preference rats.

## 2. Materials and Methods

### 2.1. Experimental Animals and Drugs

Male Sprague-Dawley rats (12-weeks-old, 200–220 g) were housed individually in plastic cages under controlled temperature (21–23°C), humidity (50%), and a 12 h/12 h light/dark cycle (light on at 0800) with access to chow and water ad libitum. The rats were purchased from the Laboratory Animal Center of Hebei Medical University. The experimental procedures followed the Guide for the Care and Use of Laboratory Animals (National Research Council, 1996) and were approved by the Animal Care and Use Committee of Hebei Medical University. All rats were randomly divided into the control (*n* = 36) and fructose preference groups (*n* = 36).

Rats were habituated to a limited period of access to water before the experiment. They were allowed to drink water from 0930 to 1130 and from 1600 to 1700 to assure that the rats obtained a daily physiological requirement of water during later experiments when they accessed the testing solution at the given time.

The solutions of lithium chloride (MP Biomedicals, Shanghai Co., 0.15 M/L), fructose (Biotopped, 10% W/W), saccharine (Fluka Chemie, Germany, 0.1% W/W), and glucose (Beichen Fangzheng, Tianjin Co., 10% W/W) were prepared in deionized water, and the test solution preference was conducted in the rat's home cage [17].

### 2.2. Fructose Preference Test

The conditioned and unconditioned procedures were similar to the procedure in a previous study with minor modifications [18]. The rats began fasting daily from 0800 h and were simultaneously administered with lithium chloride (0.15 M at 1.0 mL/100 g body weight, intraperitoneal injection). One and a half-hour later, the rats were allowed to access 10% (*w*/*v*) fructose solution for 2 h (0930–1130 h), with the total intake of the solution measured. The rats fasted in a 3.5 h induction period. During the other 20.5 h in the day, food was provided ad libitum. This procedure was performed daily for 10 consecutive days.

### 2.3. Preference Tests

Before each of the following tests, rats were deprived of water for 12 h.

#### 2.3.1. Fructose Preference Test

Prior to the fructose preference test, all rats were exposed to 10% fructose solution for 15 min to prevent neophobia [19]. All rats were allowed to adapt to drinking water in the two bottles (1600–1700 h) for two days. In the two-bottle test, rats were provided one bottle with 10% fructose solution and simultaneously another bottle with tap water for 30 min [20]. The fructose solution preference ratio was calculated as follows [21]: fructose solution preference ratio = [fructose solution intake/(fructose solution intake + water intake)] × 100%.

#### 2.3.2. Fructose Concentration Gradient Test

The rats in the fructose preference and control groups were given 10%, 8%, 6%, 4%, and 2% concentration fructose solutions, respectively. The tests were performed in the morning (0930) for 2 h every day for 5 successive days.

#### 2.3.3. Saccharine and Glucose Choice Tests

The one-bottle test was conducted in all rats. In this test, rats received one bottle of 0.1% saccharine solution for 2 h in the morning, then the next day a 10% glucose solution with all volumes of intake was recorded.

The two-bottle test for saccharine or glucose preference was conducted in fructose preference rats. Rats received one bottle of 0.1% saccharine solution and one bottle of 10% glucose solution at the same time. To avoid the interference of the flavor tastes of saccharine and glucose, this test was repeated by using these solutions with an addition of 0.1% grape flavor to ensure they have the same flavor.

### 2.4. Tissue Preparation and Neuropeptide Western Blotting

The intake volumes of water, 0.1% saccharine, and 10% glucose solutions were controlled in the metabolic monitoring system (CLAMS; Columbus, OH, USA) at 0900. The solution volumes were given as 2 ml/100 g body weight [22, 23]. After 30 min of solution intake, rats were sacrificed by an overdose of pentobarbital sodium (60 mg/kg). The whole brain was immediately removed and placed on a cold rat brain matrix. The LH and NAc were microdissected [24, 25] according to the rat brain atlas [26]. A 1.0 mm coronal slice was taken from a bregma of 1.70 mm to 0.60 mm for the NAc and a bregma of −1.80 mm to −3.80 mm for the LH [27, 28]. Tissue samples were obtained bilaterally for the LH or NAc, respectively. The tissue was finally snap frozen in liquid nitrogen and stored at −80°C for Western blot analysis. The POMC polyclonal antibody (1 : 1000, Bioworld BS7477) was used to detect endogenous levels of the POMC protein and its cleavage products in the NAc and LH, according to standard operating procedures, as described previously [29].

### 2.5. Data Presentation and Statistical Analysis

Data are presented as mean ± SEM. Statistical analyses were performed by using an SPSS version 19 (IBM Institute Inc., Armonk, NY, USA). Data obtained from the tests in the study were analyzed using Student *t*-test or one-way ANOVA with a post hoc Tukey's test to compare the data from multiple groups. Statistical significance was set at *p* < 0.05.

## 3. Results

### 3.1. Fructose Preference Rats Displayed Greater Preference for Glucose or Fructose Solutions than Control Rats

**Figure 1:**
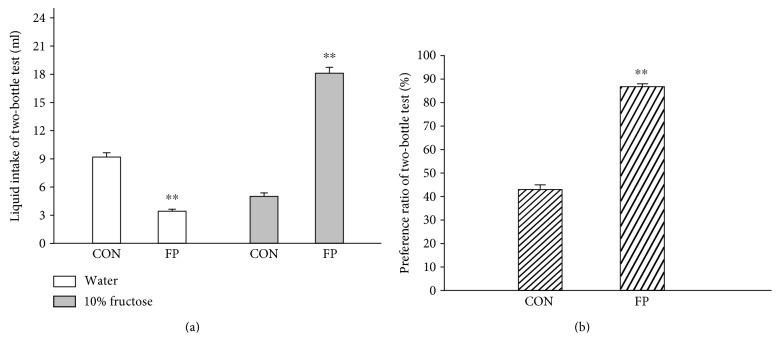
Two-bottle test for fructose preference (liquid intake (a) and preference ratio (b)). Data are represented as mean ± SEM, *n* = 15 in each group. ^∗∗^*p* < 0.01 control rats (CON) versus fructose preference rats (FP).

Two-bottle tests were used to measure the preference for fructose solution on fructose preference and control rats (Figure 1). The body weights of the fructose preference rats did not significantly differ from the control rats (data not shown). There were significant differences in the intake of the glucose solution (*p* < 0.01) and the fructose solution preference ratio between the two groups (*p* < 0.01).

**Figure 2:**
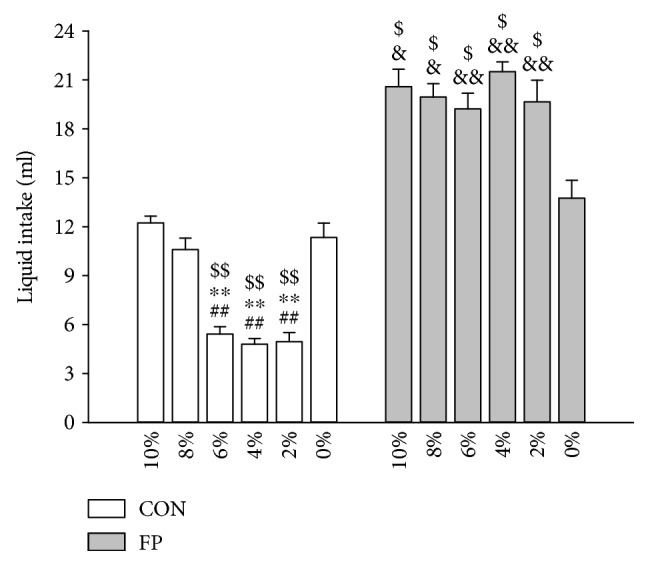
Fructose concentration gradient test. Data are represented as mean ± SEM, *n* = 8 in each group. ^∗∗^*p* < 0.01 versus 10% fructose solution, ^##^*p* < 0.01 versus 8% fructose solution, ^$^*p* < 0.05, and ^$$^*p* < 0.01 versus 0% fructose solution intragroup and ^&^*p* < 0.05 and ^&&^*p* < 0.01 control rats (CON) versus fructose preference rats (FP) for corresponding concentration of fructose solution.

In the fructose concentration gradient test, 10%, 8%, 6%, 4%, and 2% fructose solutions were given for 2 h (Figure 2). The intake volumes of each concentration of solution did not differ in the fructose preference rats at low concentrations (even at 2% concentration). The fructose solution intake at 6% concentration was significantly lower than that of 10% (*p* < 0.01) and 8% in control rats (*p* < 0.01). The intake volumes for each concentration from 10% to 2% of fructose solution were higher in fructose preference rats than the respective concentrations of fructose solutions in control rats (Figure 2). These results suggest that fructose preference rats have a higher preference for fructose solution than control rats.

### 3.2. Saccharine and Glucose Choice Tests

**Figure 3:**
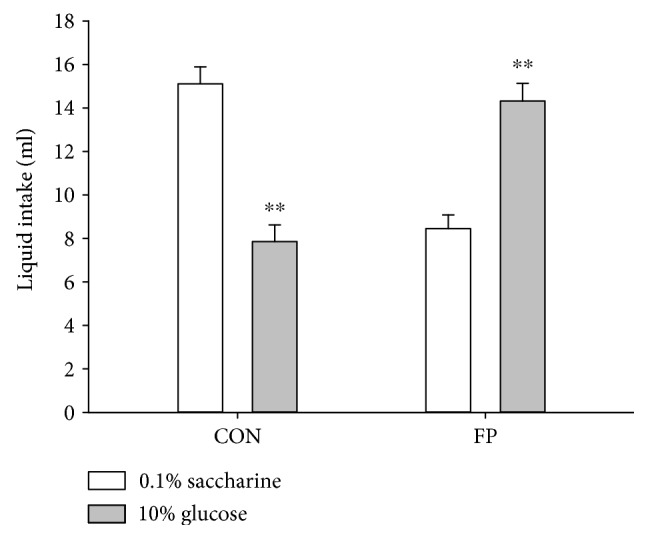
One-bottle test for saccharine and glucose choice. Data are represented as mean ± SEM, *n* = 13 in each group. ^∗∗^*p* < 0.01 versus corresponding 0.1% saccharine.

**Figure 4:**
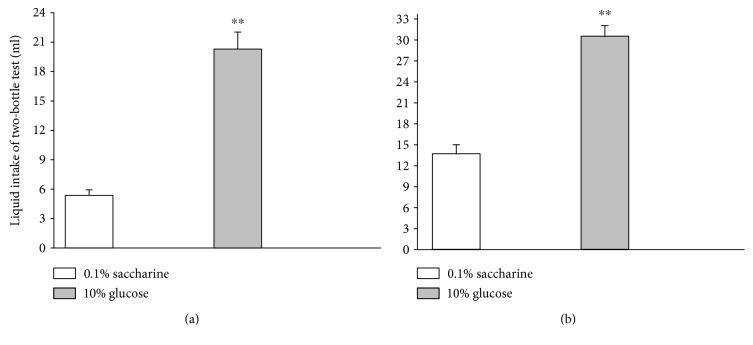
Two-bottle test for saccharine and glucose choice (2 h intake test (a) and flavor solution test (b) of fructose preference rats). Data are represented as mean ± SEM, *n* = 15 in each group. ^∗∗^*p* < 0.01 10% glucose versus 0.1% saccharine.

To determine the elements (sweet taste or calories) in fructose that are involved in glucose preference in fructose preference rats, one-bottle choice test was conducted with 0.1% saccharine solution and 10% glucose solution (Figure 3). Fructose preference rats preferred glucose solution (*p* < 0.01), while control rats preferred saccharine solution (*p* < 0.01). The two-bottle choice test revealed that the intake volume of glucose solution was higher than saccharine solution in fructose preference rats (*p* < 0.01) (Figure 4(a)). To avoid the possible interference of flavor taste on the preference to these two solutions, a new spicy flavor (grape flavor) was added into the solutions to normalize the flavor taste of saccharine and glucose solutions. Fructose preference rats showed higher intake volumes for glucose than saccharine solution (*p* < 0.01, Figure 4(b)). These findings suggest that the fructose preference rats prefer calories with sweet taste than noncalories with sweet taste.

### 3.3. Expression of Neuropeptides in the LH and NAc

**Figure 5:**
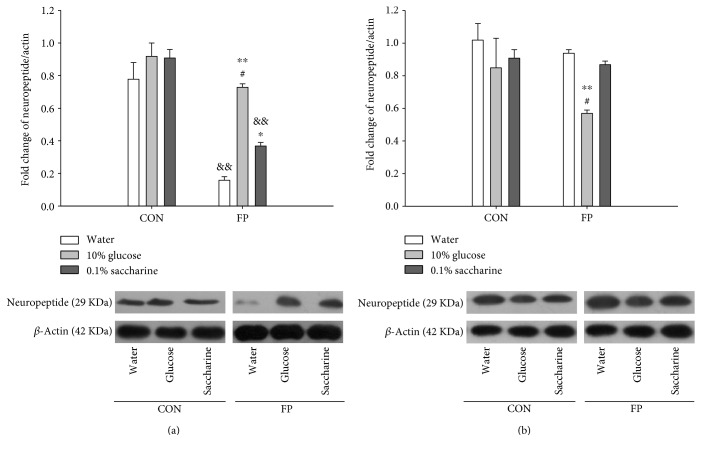
Expression of the POMC protein in the NAc (a) and LH (b) of rats intervened with water, 0.1% saccharine, and 10% glucose. Data are represented as mean ± SEM, *n* = 3 in each group. ^∗^*p* < 0.05 and ^∗∗^*p* < 0.01 versus water intragroup, ^#^*p* < 0.05 versus 0.1% saccharine intragroup, and ^&&^*p* < 0.01 control rats (CON) versus fructose preference rats (FP) for corresponding solution.

The Western blot results showed that there were no significant differences in the expression of neuropeptides derived from POMC in the NAc of control rats exposed to three kinds of solution stimuli. The expression of neuropeptides in the NAc of fructose preference rats with glucose solution was increased than that of water and 0.1% saccharine solutions (*p* < 0.01 and *p* < 0.05). Also, the 0.1% saccharine solution increased the expression of the neuropeptides compared with water (*p* < 0.05) (Figure 5(a)). Compared with control rats in the corresponding solution, the expression of neuropeptides in the NAc of fructose preference rats decreased in water (*p* < 0.01) and 0.1% saccharine solutions (*p* < 0.01).

The expression of neuropeptides derived from POMC was decreased in the LH in fructose preference rats fed by 10% glucose compared with rats fed by water or 0.1% saccharine solutions (*p* < 0.05). The expression of neuropeptides derived from POMC showed no difference in control rats fed by 10% glucose, water, or 0.1% saccharine solutions (Figure 5(b)).

## 4. Discussion

In this study, fructose preference rats were established by using the backward conditioning procedure, in which fructose solution was given following lithium chloride injection. This unconditioned solution-conditioned solution pairing tends to endow the conditioned solution (fructose) to promote the preference learning of rats [14]. Further tests observed that fructose preference rats drunk more low-concentration (2%) fructose solution than control rats, suggesting that fructose preference rats prefer nonsweet glucose solution [30]. We found that the expression level of neuropeptides derived from POMC in the LH and NAc of fructose preference rats was changed by glucose feeding, but not by saccharine intake. The POMC protein and the neuropeptides derived from POMC include *α*-MSH, *β*-MSH, ACTH, *β*-END, and *β*-LPH. These data suggest that neuroplasticity that occurs in the LH and NAc may be involved in the preference of calorie-containing sweet solution in fructose preference rats. In addition, the expression levels of neuropeptides derived from POMC are significantly decreased in fructose preference rats than in the control rat group. It is possible that POMC and the neuropeptides derived from POMC had distinct functions or sensitivities to glucose in different nuclei.

These fructose preference rats strongly prefer fructose solution and binge drinking a large amount of fructose solution in a short period of time in the two-bottle test, revealing some features of binge eating and preference [31, 32]. Rats are not only attracted to the sweet taste of sugar but they also acquire a preference for flavors associated with calories of sugar. Saccharine is a classic artificial sweetener that contains no calories. Rats may develop conditioned flavor preferences due to the sweet taste of saccharine [16]. On the other hand, fructose contains calories, has postoral actions, and causes flavor preference in rats. Similarly, glucose is a monosaccharide and is very effective in supporting postoral flavor conditioning [16]. It has been shown that mice lacking the sweet taste receptors are initially not able to recognize diluted glucose solution and strongly prefer concentrated solutions [33]. The sweet taste receptor knockout mice develop a preference for high concentrations of sugar solution demonstrating that they prefer sugar calorie more than sweet taste. Consistently, we found in this study that fructose preference rats prefer 10% glucose to 0.1% saccharine. Taken together, the results demonstrate that rats have a preference for calorie-containing sugar. This preference may be due to complex neuroplasticity of neuronal circuits involving the POMC system in the LH and NAc.

Previous studies have found that glucose levels in the rat brain increase 30 min after food intake [34]. Furthermore, a small amount of preloaded sucrose for 30 min in rats markedly changes the expression level of neuropeptides in the ARC [35]. We found that in fructose preference rats, glucose solution intake increased the expression of neuropeptides derived from POMC in the NAc, whereas it decreased the expression of these neuropeptides in the LH. However, saccharine solution intake had little effect on the expression of these neuropeptides. The data suggests that glucose preference is associated with neuroplasticity in the brain reward system. Previous studies have shown that the NAc to the LH pathway that is involved in food reward contributes to hedonic feeding or overconsumption of palatable calorie-dense food [36]. The neuroanatomical circuits from the NTS to the NAc and from the ARC to the LH indicate that the POMC system forms a complex neural circuit to regulate feeding behavior [6, 37]. On the other hand, findings from this study suggest that glucose preference leads to an alteration of POMC neural circuits (at least a change of the expression levels of neuropeptides derived from POMC).

POMC is synthesized from precursor pre-proopiomelanocortin (pre-POMC) and can be cleaved to generate multiple neuropeptides, which are involved in many neurological functions including food addiction [38]. We realized that a limitation exists in this study. The antibody used to detect multiple peptides derived from POMC was generated using full-length human POMC as an immunogen (according to the manufacturer's instruction). Therefore, this antibody should detect ACTH and *β*-LPH, which typically originate from adenohypophysis (anterior pituitary gland). In fact, POMC neurons in the ARC of the hypothalamus and the NTS express prohormone convertase 2, which cleaves ACTH and *β*-LPH to generate *α*-MSH/CLIP and *γ*-LPH/*β*-MSH/*β*-endorphin, respectively [9]. It is possible that these “POMC-derived” peptides found in the NAc and the LH may have originated from brain regions that produce POMC precursor protein. Thus, changes in homeostasis and food addiction as well as changes in the neuroendocrine system such as the hypothalamus-pituitary-adrenal gland neuroendocrine axis may be involved in this fructose preference phenotype. It is possible that POMC functions as a “switch” to control the choice of caloric-containing foods. Further studies may be needed to clarify the role of POMC and its products in different brain nuclei in feeding behavior.

In summary, we found in this study that fructose preference rats prefer glucose solution, but not saccharine solution. Glucose intake changed the expression of neuropeptides derived from POMC in the LH and NAc. The data indicates that the POMC system in the LH of the hypothalamus and the NAc of the central reward system might be involved in food preference development.
